# HTLV‐1 Tax‐specific memory cytotoxic T lymphocytes in long‐term survivors of aggressive‐type adult T‐cell leukemia/lymphoma

**DOI:** 10.1002/cam4.4689

**Published:** 2022-03-22

**Authors:** Tatsuro Jo, Kazuhiro Noguchi, Takahiro Sakai, Ritsuko Kubota‐Koketsu, Sadaharu Irie, Masatoshi Matsuo, Jun Taguchi, Kuniko Abe, Kazuto Shigematsu

**Affiliations:** ^1^ Department of Hematology Japanese Red Cross Nagasaki Genbaku Hospital Nagasaki Japan; ^2^ Department of Laboratory Japanese Red Cross Nagasaki Genbaku Hospital Nagasaki Japan; ^3^ Department of Viral Infections, Research Institute for Microbial Diseases Osaka University Osaka Japan; ^4^ Department of Pharmacy Japanese Red Cross Nagasaki Genbaku Hospital Nagasaki Japan; ^5^ Department of Pathology Japanese Red Cross Nagasaki Genbaku Hospital Nagasaki Japan

**Keywords:** adult T‐cell leukemia/lymphoma, cytotoxic T‐lymphocytes, herpes virus infection, human T‐cell lymphotropic virus type 1, Tax

## Abstract

**Purpose:**

Adult T‐cell leukemia/lymphoma (ATLL) is a relatively refractory peripheral T‐cell lymphoma caused by human T‐cell lymphotropic virus type 1 (HTLV‐1). The objective of this study was to investigate the characteristics of long‐term survivors with ATLL.

**Methods:**

We conducted an observational study of 75 aggressive‐type ATLL patients. Flow cytometry was conducted to analyze HTLV‐1 Tax‐specific cytotoxic T‐lymphocytes (CTLs) and T‐cell receptor Vβ gene repertoire.

**Results:**

We first evaluated six long‐term survivors among 37 patients who were newly diagnosed with ATLL and then treated with intensive chemotherapy without mogamulizumab, a monoclonal antibody for C‐C chemokine receptor four antigen. Reversal of the CD4‐to‐CD8 ratio (CD4/CD8) in peripheral mononuclear cells was observed in all six patients. Three of these six patients showed reversed CD4/CD8 immediately after herpes virus infection. Four of these six patients who could be examined demonstrated long‐term maintenance of HTLV‐1 Tax‐specific CTLs. We subsequently identified four long‐term survivors among 38 patients who were newly diagnosed with ATLL and then treated with intensive chemotherapy plus mogamulizumab. All four patients showed reversed CD4/CD8, and three of the four patients contracted herpes virus infection during immunochemotherapy. Six of the total 10 patients were subjected to CTL analyses. Tax‐specific CTLs were observed, and the CTLs that were almost entirely composed of memory CTLs in all patients were recorded. HTLV‐1 provirus was also detected in all six patients.

**Conclusions:**

These data suggest that Tax‐specific memory CTLs probably, together with anticancer agents, eradicate ATLL cells and exhibit long‐term preventive effects from relapse ATLL. Thus, the strong activation of cellular immunity, such as herpes virus infection, seems to be necessary to induce such a potent number of Tax‐specific CTLs.

## Graphical abstract

Long‐term survivors with complete remission of newly diagnosed aggressive‐type adult T‐cell leukemia/lymphoma were investigated clinically and immunologically. HTLV‐1 Tax‐specific memory cytotoxic T lymphocytes were observed in all survivors. Strong activation of cellular immunity, such as herpes virus infection during or immediately after chemotherapy, may be necessary to induce such a potent number of Tax‐specific cytotoxic T lymphocytes.

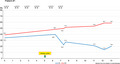

## 1 INTRODUCTION

Adult T‐cell leukemia/lymphoma (ATLL) is a CD4‐positive peripheral T‐cell lymphoma and the causative agent of human T‐cell lymphotropic virus type 1 (HTLV‐1).^1^, ^2^, ^3^, ^4^ ATLL can be classified into four subtypes: acute, lymphoma, smoldering, and chronic.^5^ The prognosis of aggressive‐type ATLL consisting of acute, lymphoma, and chronic type with poor prognostic factors is dismal.^6^ Allogeneic hematopoietic stem cell transplantation (allo‐HSCT) can be considered if the patients with aggressive‐type ATLL can achieve complete response (CR) with the first‐line of chemotherapy.^7^ HTLV‐1 Tax‐specific cytotoxic T lymphocytes (CTLs) have been reported in some patients with ATLL who were successfully treated with allo‐HSCT.^8^, ^9^ Mogamulizumab, a monoclonal antibody for C‐C chemokine receptor 4 antigen, and lenalidomide, an immunomodulating agent, have recently been approved for use in the treatment of newly diagnosed and relapsed/refractory aggressive‐type ATLL.^10^, ^11^, ^12^ Patients treated with mogamulizumab often experience mogamulizumab‐induced skin disorders with the infiltration of CD3‐ and CD8‐positive T‐cells.^13^, ^14^ We recently reported that Tax‐specific CTLs are often observed in newly diagnosed patients with aggressive‐type ATLL and mogamulizumab‐induced skin disorders. Interestingly, a statistically significant overall survival benefit was observed in these patients than in those without mogamulizumab‐induced skin disorders and under the combination therapy of mogamulizumab and intensive chemotherapy such as VCAP‐AMP‐VECP (mLSG15) and etoposide, vincristine, doxorubicin, cyclophosphamide, and prednisolone (EPOCH).^14^ However, in a phase‐II study comparing treatment outcome with mogamulizumab plus mLSG15 and with mLSG15 alone, no significant difference was noted in the overall survival and progression‐free survival between the two treatments arms, albeit the combination arm showed a better overall response rate.^11^ These results may be attributed to the fact that mogamulizumab was designed to activate natural killer (NK) cells rather than CTLs.^15^ These data suggest the importance of antitumor cellular immunity, especially CTLs, in treating aggressive‐type ATLL. It has been reported that dormant tumor lesions in immunocompetent mice treated with methylcholanthrene, a tar component, developed into progressive tumors after the depletion of T lymphocytes.^16^ This phenomenon was not observed when the NK cells were depleted. These results indicate the importance of T‐cell immunity as an acquired immune system toward controlling cancer progression. To investigate the clinical and immunological features of long‐term survivors with complete remission of newly diagnosed aggressive‐type ATLL that was treated with intensive chemotherapy with and without mogamulizumab, we analyzed the T lymphocytes of these patients, with a focus on HTLV‐1 Tax‐specific CTLs.

## 2 PATIENTS AND METHODS

### 2.1 Patient eligibility

This was a single institution observational study conducted on subjects aged ≥20 years, who were newly diagnosed with aggressive‐type ATLL. All patients included in this study were ineligible for allo‐HSCT and were treated at our institute.

We examined 75 patients, including 37 patients, who received intensive chemotherapy between July 2000 and October 2016, and 38 patients, who received intensive chemotherapy plus mogamulizumab between December 2010 and December 2018.

### 2.2 Treatment

Patients were treated with intensive chemotherapy with or without mogamulizumab. The mLSG15, CHOP, and EPOCH therapies were applied as the intensive chemotherapy regimens.^11^, ^14^, ^17^ The mLSG15 regimen consisted of the following three regimens: VCAP (vincristine, 1 mg/m^2^, maximum 2 mg; cyclophosphamide, 350 mg/m^2^; doxorubicin, 40 mg/m^2^; and prednisolone, 40 mg/m^2^) at day 1; AMP (doxorubicin, 30 mg/m^2^; ranimustine, 60 mg/m^2^; and prednisolone, 40 mg/m^2^) at day 8; and VECP (vindesine, 2.4 mg/m^2^ at day 15; etoposide, 100 mg/m^2^ on days 15–17; carboplatin, 250 mg/m^2^ at day 15; and prednisolone, 40 mg/m^2^ on days 15–17). The CHOP regimen consisted of doxorubicin (50 mg/m^2^ at day 1), cyclophosphamide (750 mg/m^2^ at day 1), vincristine (1.4 mg/m^2^, maximum 2 mg, at day 1), and prednisolone (40 mg/m^2^ on days 1–5). The EPOCH regimen consisted of continuous intravenous infusions conducted on days 1–4 with etoposide (50 mg/m^2^), vincristine (0.4 mg/m^2^), and doxorubicin (10 mg/m^2^), with bolus doses of cyclophosphamide (750 mg/m^2^ at day 6) and oral prednisolone (60 mg/m^2^ on days 1–6). We recently reported a risk reduction of approximately 30% in patients treated with mogamulizumab + EPOCH compared to those treated with mogamulizumab + mLSG15.^14^

### 2.3 Immunological analysis and HTLV‐1 provirus load

Peripheral blood mononuclear cells (PBMCs) were collected from patients with human leukocyte antigen (HLA)‐A*02:01 and/or HLA‐A*24:02. Tax‐specific CTLs were determined by flow cytometry without any stimulation. HLA‐A*02:01‐restricted Tax tetramer (HTLV‐1 Tax 178–186 Peptide, MBL) and HLA‐A*24‐02‐restricted Tax tetramer (HTLV‐1 Tax 301–309 Peptide, MBL) were used for detecting Tax‐specific CTLs. The analyses were performed by SRL Corporation and at our institution. Tax‐specific CTLs were further analyzed to categorize them into memory, effector, and naïve CTLs by flow cytometry using antibodies for CD27 and CD45RA (Beckman Coulter). Memory CD8‐positive T lymphocytes were defined as the CD8^+^CD27^+^CD45RA^−^ population, effector CD8‐positive T lymphocytes as the CD8^+^CD27^−^CD45RA^+^ population, and naïve CD8‐positive T lymphocytes as the CD8^+^CD27^+^CD45RA^+^ population.

Tax‐specific CTLs have been further analyzed for T‐cell receptor (TCR) Vβ gene repertoire by flow cytometry using the Beta Mark TCR Vβ Repertoire Kit (Beckman Coulter) according to the manufacturer's instructions. This kit consisted of Kit‐A for TCR *Vβ 5.3*, *Vβ 7.1*, and *Vβ 3*; Kit‐B for TCR *Vβ 9*, *Vβ 17*, and *Vβ 16*; Kit‐C for TCR *Vβ 18*, *Vβ 5.1*, and *Vβ 20*; Kit‐D for TCR *Vβ 13.1*, *Vβ 13.6*, and *Vβ 8*; Kit‐E for TCR *Vβ 5.2*, *Vβ 2*, and *Vβ 12*; Kit‐F for TCR *Vβ 23*, *Vβ 1*, and *Vβ 21.3*; Kit‐G for TCR *Vβ 11*, *Vβ 22*, and *Vβ 14*; and Kit‐H for TCR *Vβ 13.2*, *Vβ 4*, and *Vβ 7.2*.

The percentages of CD4‐ and CD8‐positive T lymphocytes in the PBMCs were analyzed by flow cytometry at several time points during chemotherapy and the follow‐up period.

To quantify HTLV‐1 provirus load, genomic DNA extracted from the samples were analyzed using real‐time polymerase chain reaction, as previously described.^18^ The HTLV‐1 provirus load was determined at the same time when Tax‐specific CTLs were analyzed by SRL Corporation.

**FIGURE 1:**
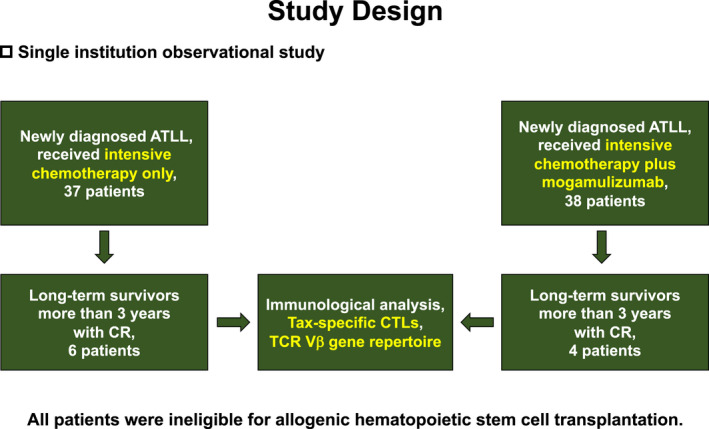
Flow diagram illustrating the study design. ATLL, adult T‐cell leukemia/lymphoma; CR, complete remission; TCR Vβ, T‐cell receptor V beta

Detailed immunological examinations were performed in 10 of the total 75 patients. Written informed consent was obtained from all of the 10 patients for their participation in this study. The study protocol was approved by the Institutional Ethical Review Board and was conducted in accordance with the Declaration of Helsinki and its later amendments. The study design has been illustrated in Figure 1.

## 3 RESULTS

### 3.1 Long‐term survivors treated with intensive chemotherapy without mogamulizumab

We first evaluated 37 patients who were newly diagnosed with aggressive‐type ATLL and then treated with intensive chemotherapy alone (Table 1). A total of 34 patients were treated with mLSG15 and three were treated with a CHOP‐like regimen. Of these, six patients achieved long‐term survival with CR. Patients #1, #2, #3, #4, and #6 survived for >10 years, but patient #5 died due to secondary chronic myelomonocytic leukemia without ATLL relapse. The overall survival of patient #5 from the time of initiation of chemotherapy was 71 months.

**TABLE 1:** Long‐term survivors with complete remission who were initiated on intensive chemotherapy between July 2000 and October 2016

Treatment regimen	N
VCAP‐AMP‐VECP (mLSG15)	34
CHOP‐like	3

Number of patients	Sex	Age at diagnosis	Subtype of ATLL	Survival after starting chemotherapy (months)	CD4/CD8 reversal	Herpes virus infection	HLA
1	Male	69	Lymphoma	>221	+	+	A*02:01
2	Male	68	Lymphoma	>211	+	+	A*02:01
3	Female	62	Lymphoma	>177	+	−	A*02:01 A*24:02
4	Female	61	Acute	>125	+	−	A*02:01 A*24:02
5	Female	71	Lymphoma	71	+	−	N.E.
6	Male	60	Lymphoma	>192	+	+	N.E.

Abbreviations: ATLL, adult T‐cell leukemia/lymphoma; HLA, human leukocyte antigen; N.E., not examined.

**FIGURE 2:**
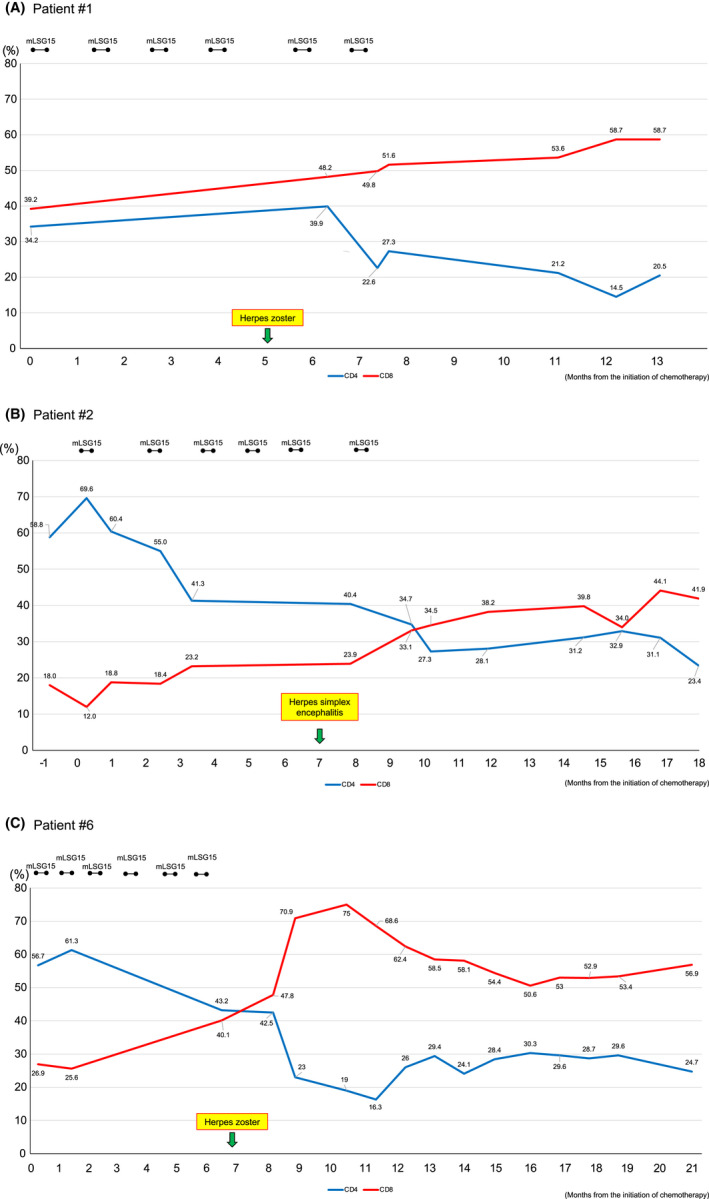
Changes in the levels of CD4‐ and CD8‐positive T‐lymphocytes in three aggressive‐type ATLL patients treated with intensive chemotherapy alone. (A) Patient #1; (B); Patient #2; and (C) Patient #6. The percentages of CD4‐positive (blue lines) and CD8‐positive (red lines) T‐lymphocytes are indicated. Black bars indicate VCAP‐AMP‐VECP (mLSG15) chemotherapy. The occurrences of herpes virus infection are indicated with arrows

The reversal of the CD4‐to‐CD8 ratio (CD4/CD8) in PBMCs was noted during chemotherapy for all long‐term survivors. Three of the survivors (i.e., patients #1, #2, and #6) contracted herpes virus infection after obtaining CR during chemotherapy or the completion of chemotherapy. In patient #1, the ratio of CD8‐positive T lymphocytes was higher than that of CD4‐positive T lymphocytes before starting with chemotherapy, but the difference increased after the patient developed herpes zoster (Figure 2A). In patients #2 and #6, the CD4/CD8 reversal was immediately observed after the onset of herpes simplex encephalitis and herpes zoster, respectively (Figure 2B,C).

**FIGURE 3:**
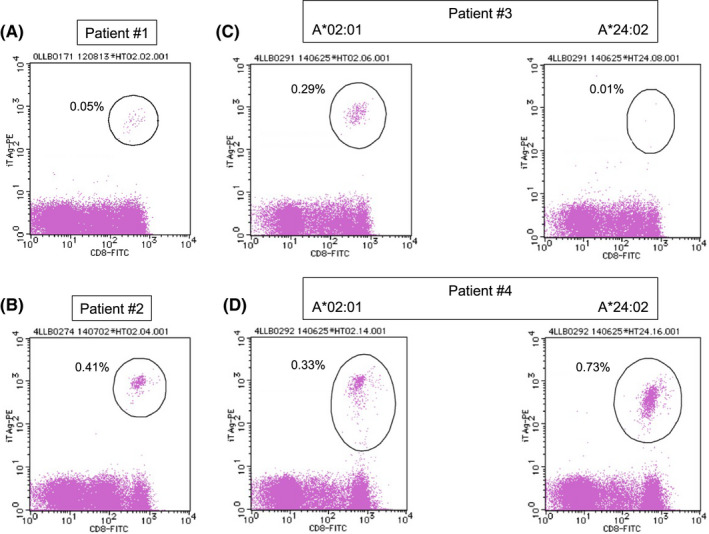
HTLV‐1 Tax‐specific CTLs in four patients with aggressive‐type ATLL treated with intensive chemotherapy alone. (A) Patient #1; (B) Patient #2. (C) Patient #3; and (D) Patient #4. The percentages of Tax‐specific CTLs in lymphocytes are indicated. The dots surrounded by the circles indicate Tax‐specific CTLs

Patients #1 and #2 had HLA‐A*02:01, whereas patients #3 and #4 both showed HLA‐A*02:01 and HLA‐A*24:02. These four patients were subjected to Tax‐specific CTL analysis twice after the completion of the chemotherapy regimen (Table 2). Tax‐specific CTLs were detected in all patients. The representative flow cytometry data are presented in Figure 3. Although these analyses were performed without any stimulation, the percentage of Tax‐specific CTLs in the lymphocytes was extremely high in all four patients (0.05%–0.73%), except for HLA‐A*24:02‐restricted CTLs in patient #3 (0.01%). Although all four patients maintained CR at each examination time, HTLV‐1 provirus remained detectable in small quantities (7.1–61.9 copies/1000 PBMCs) (Table 2).

**TABLE 2:** Tax‐specific CTLs and HTLV‐1 provirus load in the peripheral blood samples

Number of patients	Date of examination from starting chemotherapy (months)	HLA	Tax‐specific CTLs/lymphocyte (%)	HTLV‐1 provirus DNA (copies/1000PBMCs)
1	133	A*02:01	0.05	35.4
	155		0.05	61.9
2	118	A*02:01	0.23	24.4
	129		0.41	14.7
3	84	A*02:01	0.26	7.1
		A*24:02	0.01	
	104	A*02:01	0.29	13.3
		A*24:02	0.01	
4	31	A*02:01	N.E.	26.4
		A*24:02	0.64	
	53	A*02:01	0.33	27.9
		A*24:02	0.73	

Abbreviations: CTLs, cytotoxic T lymphocytes; HLA, human leukocyte antigen; HTLV‐1, human T lymphotropic virus type 1; N.E., not examined; PBMCs, peripheral blood mononuclear cells.

### 3.2 Long‐term survivors treated with intensive chemotherapy with mogamulizumab

We next investigated 38 patients who were newly diagnosed with aggressive‐type ATLL and then treated with intensive chemotherapy with mogamulizumab (Table 3). Of these, 22 patients were treated with mogamulizumab plus EPOCH, 14 with mogamulizumab plus mLSG15, and two with mogamulizumab plus a CHOP‐like regimen. Of these 22 patients, four achieved long‐term survival with CR. CD4/CD8 reversal was observed after the first cycle of immunochemotherapy in each patient, mainly due to the effect of mogamulizumab. Three of the four patients contracted herpes virus infection (herpes zoster for patient #7 and herpes simplex for patients #8 and #9) during immunochemotherapy. Patient #7 showed both HLA‐A*02:01 and HLA‐A*24:02. Patient #8 showed HLA‐A*24:02, patient #9 showed HLA‐A*02:01, and patient #10 showed neither.

**TABLE 3:** Long‐term survivors with complete remission who were initiated on mogamulizumab plus intensive chemotherapy between December 2010 and December 2018

Treatment regimen	N
Mogamulizumab plus EPOCH	22
Mogamulizumab plus VCAP‐AMP‐VECP (mLSG15)	14
Mogamulizumab plus CHOP‐like	2

Number of patients	Sex	Age at diagnosis	Subtype of ATLL	Survival after starting chemotherapy (months)	CD4/CD8 reversal	Herpes virus infection	HLA
7	Male	78	Acute	>47	+	+	A*02:01 A*24:02
8	Female	62	Acute	>41	+	+	A*24:02
9	Female	69	Acute	>37	+	−	A*02:01
10	Female	80	Lymphoma	>55	+	+	A*26:03 A*31:01

Abbreviations: ATLL, adult T‐cell leukemia/lymphoma; HLA, human leukocyte antigen.

### 3.3 HTLV‐1 Tax‐specific CTLs and TCR repertoire analysis

**FIGURE 4:**
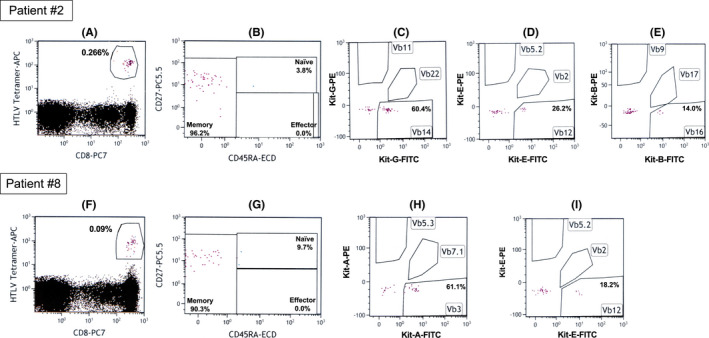
Representative data of HTLV‐1 Tax‐specific CTLs and T‐cell receptor repertoire analysis. The upper panels from (A–E) show the data on Patient #2, whereas the lower panels from (F–I) show the data on Patient #8. CD8‐positive and HTLV‐1 Tax‐specific tetramer‐positive T‐lymphocytes surrounded by the circles indicate HTLV‐1 Tax‐specific CTLs in panels (A and F). HTLV‐1 Tax‐specific memory, effector, and naïve CTLs are shown in panels (B and G). Selected T‐cell receptor Vβ gene repertoire in HTLV‐1 Tax‐specific CTLs are shown in panels (C, D, E, H, and I). Peripheral blood mononuclear cells were directly analyzed by flow cytometry without any stimulation using the HTLV‐1 Tax‐specific tetramers (BML) and the Beta Mark TCR Vβ repertoire kit (Beckman Coulter) as specified by the manufacturer

We performed a combined analysis of Tax‐specific CTLs and TCR *Vβ* gene repertoire to investigate the subdivision of memory, effector, and naïve CTLs as well as the clonality of the CTLs in patients #2, #3, #4, #7, #8, and #9 (Table 4, Figure 4). The analyses were performed after the initiation of the first antitumor treatment, and CR was maintained at the time of the analyses in all six patients (Table 4). Patients #2, #7, and #8 contracted herpes virus infection during the antitumor treatment. Tax‐specific CTLs were detected in all six patients at high percentages (0.09%–1.40%). The majority of these CTLs consisted of memory CTLs. In the HLA‐A*24:02‐restricted CTLs in patient #4, 60% were composed of memory CTLs, but, in all other cases, memory CTLs accounted for >80% (83.0%–99.8%).

**TABLE 4:** HTLV‐1 Tax‐specific CTLs, HTLV‐1 provirus DNA, and TCR repertoire analyses in the peripheral blood samples

Number of patients	HLA	Tax‐specific CTLs/lymphocyte (%)	Tax‐specific CTL subtype			TCR V beta usage major clones	HTLV‐1 provirus load (copies/1000PBMCs)	Date of examination from starting therapy (months)
			Memory (%)	Effector (%)	Naïve (%)			
2^a^	A*02:01	0.266	96.2	3.8	0.0	V beta 14: 60.4% V beta 12: 26.2% V beta 16: 14.0%	50.2	211
3	A*02:01	0.153	83.0	5.7	9.4	V beta 22: 25.0% V beta 2: 15.2%	12.3	177
	A*24:02	0.011	N.E.			Not examined		
4	A*02:01	0.308	95.7	1.1	3.2	Not determined	66.6	125
	A*24:02	0.882	61.2	0.8	38.1	Not determined		
7^a^	A*02:01	0.065	96.3	0.0	3.7	V beta 14: 43.6% V beta 3: 13.9% V beta 20: 13.6% V beta 8: 13.0% V beta 21.3: 10.5%	15.3	45
	A*24:02	0.878	99.8	0.1	0.1	Not determined		
8^a^	A*24:02	0.09	90.3	0.0	9.7	V beta 3: 61.1% V beta 12: 18.2%	137.3	39
9	A*02:01	1.40	91.7	1.7	6.5	Not determined	22.2	35

Abbreviations: CTLs, cytotoxic T lymphocytes; HLA, human leukocyte antigen; HTLV‐1, human T lymphotropic virus type 1; not determined, the usage of TCR V beta genes >10% was not detected in this study; TCR, T‐cell receptor.

^a^ Patients who experienced herpes virus infection during chemotherapy.

The Beta Mark TCR Vβ Repertoire Kit used for TCR Vβ gene repertoire analysis of Tax‐specific CTLs could be used to examine 24 TCR Vβ genes simultaneously. The usage of the TCR Vβ genes at >10% is listed in Table 4. Clonal expansion of Tax‐specific CTLs was detected in four of the six patients. In particular, >60% usage of the TCR Vβ genes was observed in patients #2 and #8. In patients #4 and #9, no clonality of TCR Vβ gene usage was detected. The representative flow cytometry data are presented in Figure 4. The percentages of Tax‐specific CTLs in lymphocytes, as indicated by circles for patients #2 and #8, were 0.266% and 0.09%, respectively (Figure 4A,F). In both the patients, >90% of Tax‐specific CTLs consisted of the memory subtype (Figure 4B,G). Selected data of the TCR *Vβ* repertoire analysis of the memory Tax‐specific CTLs are presented in Figure 4C–E,H,I. HLA‐A*02:01‐restricted memory Tax‐specific CTLs using TCR *Vβ 14* was a major clone in patient #2, whereas HLA‐A*24:02‐restricted memory Tax‐specific CTLs using TCR *Vβ 3* was a major clone in patient #8.

## 4 DISCUSSION

The main target tissues of ATLL are lymph nodes and the peripheral blood, but the skin and muscular tissues are also occasionally targeted.^19^ HTLV‐1 infects CD4‐positive T cells and causes peripheral T‐cell lymphoma. However, an occasional shift can occur in cell‐surface markers from CD4 to CD8 during the disease course in patients with ATLL.^20^ Thus, ATLL is not a straightforward disease, and its treatment is fraught with several challenges.

The various physiological activities of the Tax and HBZ proteins possibly contribute to the therapeutic difficulty of ATLL.^21^, ^22^ On the contrary, the gene sequence of HTLV‐1 is different from that of humans and hence, its gene product could possibly be recognized as a tumor antigen by host‐immunocompetent cells.^23^ However, several reports have demonstrated that ATLL cells have a high incidence of genetic and epigenetic alterations, which induces several ATLL cells to either not express Tax at all or express it at extremely low levels in the body.^24^, ^25^, ^26^, ^27^, ^28^, ^29^, ^30^ On the other hand, the detection of the *tax* mRNA expression in ATLL cells and Tax‐specific CTLs have been reported in ATLL patients, which suggests that the Tax expression in ATLL patients cannot be ignored.^8^, ^9^, ^14^, ^31^ Therefore, the clinical and immunological features, including Tax‐specific CTLs, in the long‐term survivors with CR who were affected by ATLL and treated with chemotherapy with or without mogamulizumab should be investigated.

In chemotherapy treatment conducted without mogamulizumab, we found that CD4/CD8 reversal was observed in all long‐term survivors with ATLL (Table 1). We speculated that this phenomenon could demonstrate the importance of CTLs in the treatment of ATLL. However, the modalities for examining Tax‐specific CTLs in routine medical practice were not available until 2012. Tax‐specific CTLs were examined once in 2012 and then once in 2014 in four long‐term survivors with ATLL. Although HTLV‐1 provirus DNA was detected in all these four patients, Tax‐specific CTLs were maintained in all cases (Table 2, Figure 3). Considering that T lymphocytes can maintain cancer dormancy in carcinogen‐induced cancer‐bearing mice,^16^ the identification of HTLV‐1 in long‐term survivors may indicate that some ATLL cells remain dormant for a long time. It is therefore possible that Tax‐specific CTLs may have worked together with anticancer drugs during the chemotherapy treatment period and may have played a role in preventing the regrowth of these dormant ATLL cells after the chemotherapy period in the long‐term survivors. Notably, the CD4/CD8 reversal was observed immediately after herpes virus infection in three patients (Table 1, Figure 2). Moreover, past studies have reported that herpes virus infection and varicella‐zoster virus reactivation can elicit a rapid and potent immune response and thereafter the activation of CTLs.^32^, ^33^, ^34^ In this process, antigen‐presenting cells (APCs) also get activated, which in turn may enhance the ability to present Tax peptides to CD8‐positive T cells.^8^, ^9^ Although patient #4 was not infected with a herpes virus, the patient was treated with EPOCH therapy with PEGylated interferon α2b drug after the relapse of ATLL. In general, achieving CR and long‐term survival is extremely difficult in relapsed cases of ATLL. However, patient #4 survived for >10 years with Tax‐specific CTLs.^35^ Furthermore, it has been reported that the cross‐presentation of tumor antigens by dendritic cells activated by type‐I interferon is essential for the induction of antitumor immunity.^36^, ^37^ Moreover, a past study reported that interferon α, a type‐I interferon, induces the upregulation of major histocompatibility complex class‐1 molecule on tumor cells as well as activates CTLs, which in turn facilitates the recognition of tumor cells by CTLs.^38^ Therefore, the application of interferon α may have contributed to long‐term survival for patient #4 by activating APCs and Tax‐specific CTLs. As a future challenge, incorporating interferon α into the treatment of ATLL to enhance host cellular immunity might be considered. In general, the identification of tumor‐specific CTLs is not simple. For example, PBMCs are cultured for approximately 14 days with any stimulation by tumor antigen peptides and interleukin 2, and then assessed.^39^ However, it remains intriguing that the Tax‐specific CTLs identified in this study were examined without any stimulation, and their values were extremely high (Table 2, Figure 3). Based on these data, we speculated that the upregulation of cellular immunity caused by fighting against herpes virus infection or interferon α treatment may have upregulated cellular immunity against Tax, which could have ultimately led to long‐term survival in these patients. In fact, the treatment of ATLL with the combination of zidovudine and interferon α with or without arsenic have been reported.^40^

In chemotherapy treatment conducted with mogamulizumab, CD4/CD8 reversal was noted in most patients in the early stage of the immunochemotherapy. This may be because almost all CD4‐positive cells express the CCR4 antigen. However, CD4/CD8 reversal does not always lead to long‐term survival. For instance, in a recent report, we demonstrated that Tax‐specific CTLs were often observed in ATLL patients with mogamulizumab‐induced skin disorders, and such patients obtained statistically longer survival than those without mogamulizumab‐induced skin disorders.^14^ The phase‐II study compared mLSG15 plus mogamulizumab combination therapy with mLSG15 monotherapy in newly diagnosed aggressive ATLL patients who did not show any survival benefit, although the CR rates were higher in the combination arm.^11^ These results suggest that CD4/CD8 reversal induced by mogamulizumab treatment alone may be insufficient to control ATLL and that the upregulation of Tax‐specific CTLs is essential for long‐term survival, as in the case of ATLL patients with mogamulizumab‐induced skin disorders. This result may be partially attributed to the fact that mogamulizumab was designed to primarily activated NK cells, and not CTLs.^15^ All four long‐term survivors who underwent intensive chemotherapy with mogamulizumab demonstrated CD4/CD8 reversal. Furthermore, three of the four patients got infected with herpes virus infection during the immunochemotherapy period (Table 3). Recently, we encountered an interesting case report related to patient #7. Patient #7 developed anaplastic lymphoma kinase‐negative anaplastic large cell lymphoma (ALCL) after achieving CR with mogamulizumab plus EPOCH therapy for aggressive ATLL. We found that the patient suffered from herpes zoster during the immunochemotherapy, after which upregulation of Tax‐specific CTLs was noted. Fortunately, the patient achieved CR for ALCL through treatment with romidepsin. The Tax‐specific CTLs were maintained at high percentages during the romidepsin therapy and until the last follow‐up without any relapse of ATLL and ALCL in this patient.^41^ These results suggest that mogamulizumab alone is insufficient to induce adequate Tax‐specific CTLs production. Herpes virus infection has been reported to activate innate and adaptive immune responses, which include the release of inflammatory cytokines and chemokines.^42^ The activation and proliferation of CD8‐positive T cells during viral infection have also been reported.^43^, ^44^ An event that elicits a strong immune response in patients, such as herpes virus infection, is believed to be necessary to induce adequate Tax‐specific CTLs. The phenomenon of bacterial infection leading to cancer regression was known empirically in the 18th century. Dr. William Coley conducted a trial in which cancer patients were injected with a mixture of bacteria including *Streptococcus pyogenes* and *Serratia marcescens* for therapeutic purposes, which led to some effective case developments.^45^ In addition, Dr. Hilton Levy and colleagues tested the use of viral products as interferon inducers in various carcinomas as anticancer immunotherapy. Of the 25 subjects tested, two (one with adult acute leukemia and another one with pediatric acute lymphoblastic leukemia) were reported to have achieved therapeutic outcomes.^46^ These results suggest that bacterial and viral infections can activate the immunity of cancer patients, and that the induced cytokines, such as tumor necrosis factor and interferon α can lead to a therapeutic effect.^47^, ^48^ In the present study, 10 of the 75 patients with aggressive ATLL were long‐term survivors, of which six were infected with herpes virus during or immediately after (immuno) chemotherapy. This is the first report on herpes virus infection that possibly activated the patient's immune system, thereby enhancing the efficacy of treatment for aggressive ATLL.

Finally, we further investigated the nature of the Tax‐specific CTLs in the six long‐term survivors with HLA‐A*02:01 and/or HLA‐A*24:02 (Table 4, Figure 4). The majority of Tax‐specific CTLs were memory phenotype, and the clonal expansion was observed in patients #2, #3, #7, and #8 by TCR *Vβ* repertoire analyses. However, the TCR *Vβ* repertoire analyses could not determine the clonal expansion of HLA‐A*02:01‐restricted and HLA‐A*24:02‐restricted Tax‐specific CTLs in patient #4, HLA‐A*24:02‐restricted Tax‐specific CTLs in patient #7, and HLA‐A*02:01‐restricted Tax‐specific CTLs in patient #9, suggesting that the clones had expanded by using other TCR *Vβ* genes that could not be identified with the kit used in the present study. The combination of these Tax tetramer assays with TCR Vβ repertoire assays in the present study is the first of its kind and has never been reported earlier. This novel combination of assays revealed that most of the Tax‐specific CTLs maintained in aggressive ATLL patients with long‐term survival and CR on intensive chemotherapy with or without mogamulizumab treatment were memory CTLs. HTLV‐1 provirus DNA was detected even in patients #7, #8, and #9 who were treated with intensive chemotherapy plus mogamulizumab. Therefore, chemotherapy with or without mogamulizumab and Tax‐specific CTLs could bring about CR and long‐term survival in aggressive ATLL, albeit it could not eradicate HTLV‐1 completely. Thus, we suggest that Tax‐specific CTLs can form memory CTLs, which in turn can be maintained to prevent the relapse of ATLL.

In summary, we investigated the clinical and immunological characteristics of long‐term survivors of aggressive ATLL who were treated with intensive chemotherapy alone or in combination with mogamulizumab. Out of the 75 patients, 10 were long‐term survivors, six of whom contracted herpes virus infection during or immediately after the chemotherapy. All survivors exhibited and maintained Tax‐specific CTLs for a long time, which were mostly composed of memory CTLs. Furthermore, residual HTLV‐1 provirus DNA was reported in all survivors despite long‐term CR. These results suggest that Tax‐specific CTLs, together with anticancer agents, eradicated ATLL cells during chemotherapy and, subsequently, became memory CTLs to function in the prevention of the relapse of ATLL. The strong activation of cellular immunity during herpes virus infection or via interferon administration may hence be necessary to induce such a sufficiently potent number of Tax‐specific CTLs. In addition, it is clear that mogamulizumab alone is insufficient to effectively induce such Tax‐specific CTLs. This study has some limitations, including it being a single‐center observational study and the small number of long‐term survivors assessed. Furthermore, the patients did not receive the same intensive chemotherapy. Nevertheless, the findings on Tax‐specific memory CTLs and herpes virus infection are intriguing and not reported earlier. Although this study was a retrospective observational study, future prospective studies are warranted to explore the possibility that herpes virus infection upregulating Tax‐specific CTLs may contribute to long‐term survival following (immuno) chemotherapy for ATLL. We believe that the development of a method to effectively activate the cellular immunity against ATLL cells will be necessary for the treatment of ATLL, in addition to immunochemotherapy.

## CONFLICT OF INTEREST

The authors declare no conflict of interest.

## AUTHOR CONTRIBUTIONS

Tatsuro Jo designed the work; collected, analyzed, and interpreted data; and drafted the manuscript. Kazuhiro Noguchi, Takahiro Sakai, Sadaharu Irie, Masatoshi Matsuo, Jun Taguchi, Kuniko Abe, and Kazuto Shigematsu collected and analyzed data. Ritsuko Kubota‐Koketsu interpreted the data and drafted the manuscript. All authors have read and approved the final version of this manuscript.

## PATIENT CONSENT STATEMENT

Written informed consent was obtained from all patients who were examined by immunological analyses and HTLV‐1 provirus load.

## PERMISSION TO REPRODUCE MATERIAL FROM OTHER SOURCES

No material from other sources was used in the drafting of this manuscript.

## CLINICAL TRIAL REGISTRATION

Not applicable.

## ETHICS STATEMENT

The study was approved by the Institutional Ethical Review Board (Approval Number: R3‐668) and was conducted in accordance with the Declaration of Helsinki and its later amendments.

## Data Availability

The data that support the findings of this study are available from the corresponding author upon reasonable request.
